# Synophyrs, curly eyelashes and *Ptyrigium colli* in a girl with Desbuquois dysplasia: a case report and review of the literature

**DOI:** 10.4076/1757-1626-2-7873

**Published:** 2009-09-15

**Authors:** Ali Al Kaissi, Klaus Klaushofer, Franz Grill

**Affiliations:** 1 Ludwig Boltzmann Institute of Osteology, at the Hanusch Hospital of WGKK and, AUVA Trauma Centre Meidling, 4th Medical DepartmentA-1140 Vienna, Heinrich Collin-Str. 30, Vienna; 2 Orthopaedic Hospital of Speising, Paediatric DepartmentA-1130 Vienna, Speisinger Str. 109Austria

## Abstract

**Introduction:**

Desbuquois dysplasia is a rare, but well described syndrome with remarkable clinical and radiographic variability ranging from mild skeletal involvement with normal intelligence to those with early fatal outcome.

**Case presentation:**

Distinctive radiographic features of Desbuquois dysplasia-typical hand type have been documented in a 3-year-old girl. Synophyrs, curly eyelashes and ptyrigium colli were additional findings.

**Conclusion:**

The phenotypic variability of Desbuquois syndrome might be an element of diagnostic confusion. However, distinctive radiographic features should urgently requiring attention and are virtually diagnostic. We report what might be the first clinical report of Desbuquois dysplasia from a consanguineous family in Austria. Unusual facial dysmorphism resembling Cornelia-De Lange syndrome and early patellar ossification were additional unreported features in connection with Desbuquois dysplasia.

## Introduction

Desbuquois dysplasia is a rare, but well described syndrome with remarkable clinical and radiographic variability ranging from mild skeletal involvement with normal intelligence to those with early fatal outcome [1-6]. There are supernumerary phalanges, characteristically situated between the metacarpal and proximal phalanx of the index finger, osteoporosis, a short narrow thorax, metaphyseal enlargement and the unique development of “monkey wrench” appearance of the proximal femur. Ossification in the carpal centres may be advanced, whereas the epiphyses of the long bones can have retarded development.

Subsequent reports of Desbuquois dysplasia divided the disorder into two subgroups based on the presence of a supernumerary ossification centres, and variable thumb changes, these are encountered in 46% of patients, being regarded as typical hand changes. 54% of cases have been described as being presented with atypical subtype changes [5].

Desbuquois dysplasia is reminiscent of Larsen syndrome, in that there is joint laxity with multiple dislocations. The eyes are prominent, the nasal bridge tends to be flat, and there can be marked micrognathia. Radiological changes are distinctive.

Our patient manifested unusual dysmorphic features of synophyrs, curly eyelashes relative prognathism and ptyrigium colli. Radiographically she illustrates the characteristic and the unique features of Desbuquois dysplasia. Parents were first degree related and this supports autosomal recessive pattern of inheritance.

## Case presentation

The child was referred to the orthopaedic department because of joint laxity and walking difficulties. The patient is of Turkish origin was a product of full term uneventful gestation. At birth weight and length were –2SD, whereas her ofc was around the 25^th^ Percentile. Pulmonary stenosis was diagnosed. The mother was a 27-year-old-gravida 1 abortus 0 married to first degree related 31-year-old man. Both parents were healthy. Hypotonia and increased joint laxity were noted in her first year of life.

Her subsequent course of development was within normal limits with exception of her motor skills. She begun to sit without support at age of 9 months, and walking was achieved at age of 18 months albeit with difficulties.

**Figure 1.**
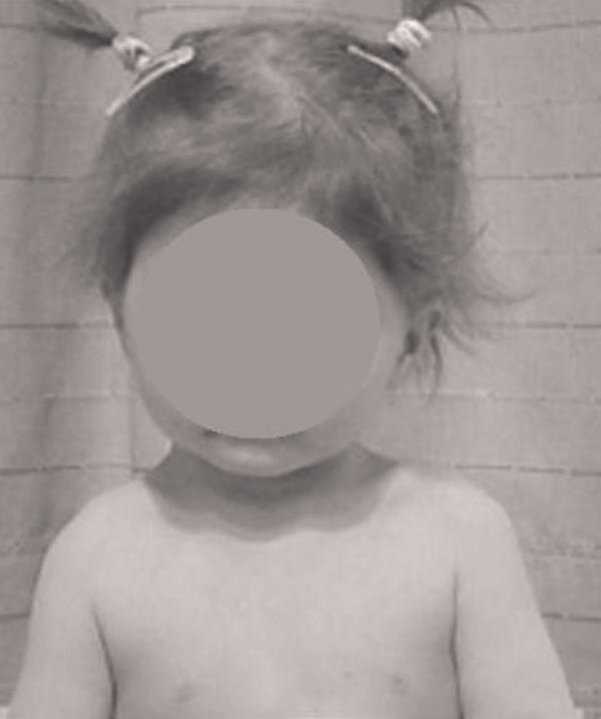
The child’s phenotype of round facies, deep-seated eyes, synophyrs, and curly eyelashes, flat nasal bridge, small nose and relative prognathism.

**Figure 2.**
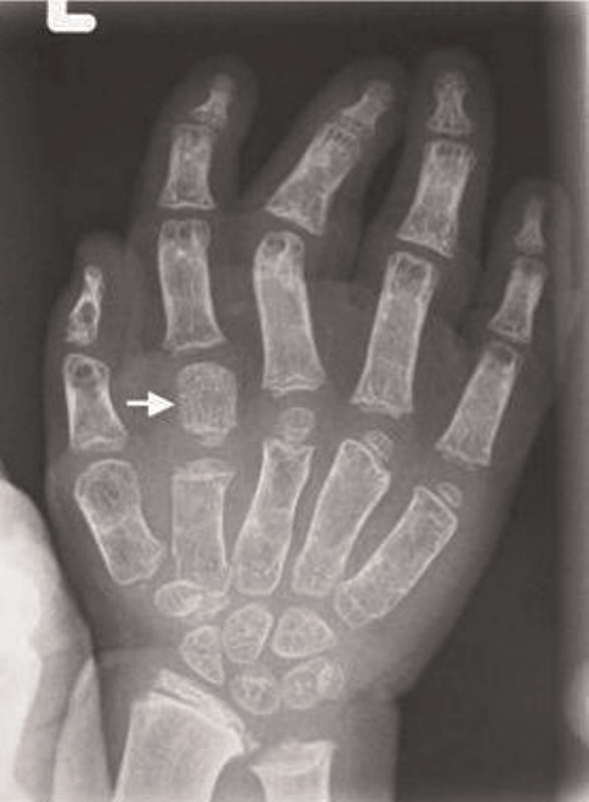
A-P radiograph of the hand showed multiple carpal ossification centres equivalent to bone age of 7 years 4 months. Supernumerary phalange characteristically situated between the metacarpal and proximal phalanx of the index finger, and existence of metaphyseal enlargement and pseudoepiphysis (arrow) were noted as well.

**Figure 3.**
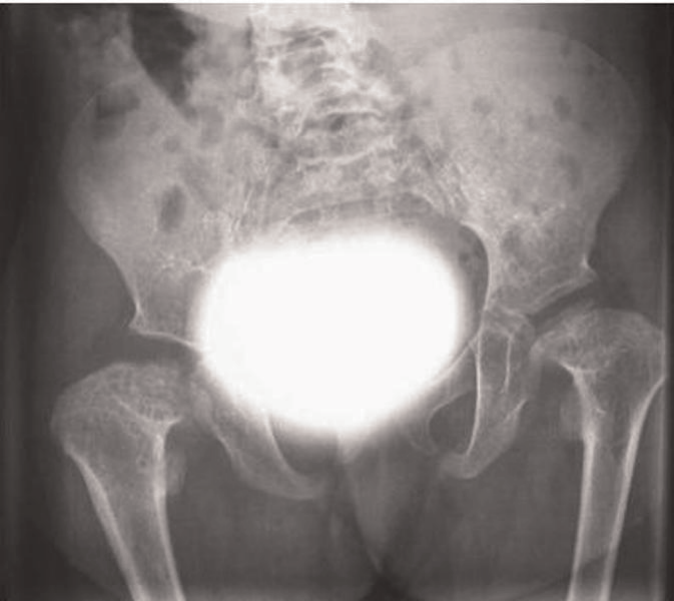
A-P radiograph of the pelvis and proximal femur showed horizontal acetabulae, coxa vara, and short femoral necks with prominence of the lesser trochanters producing the “monkey wrench” appearance of the proximal femur. Note the remarkable prominence of the lesser trochanters (arrows).

**Figure 4.**
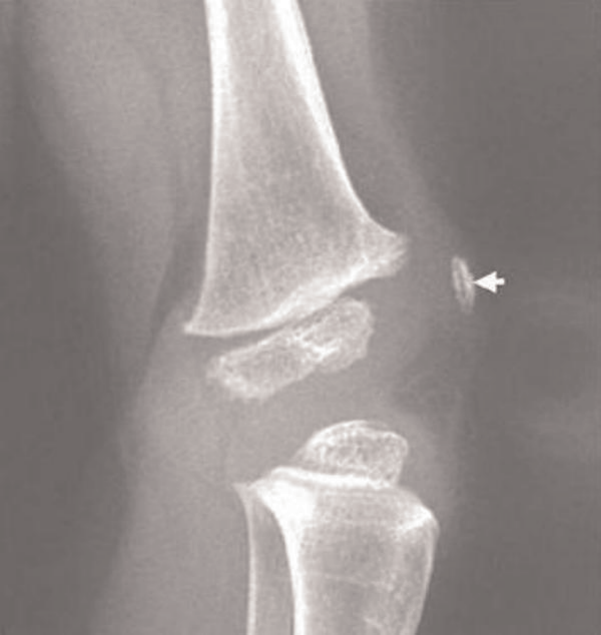
Lateral radiograph of the knee showed unusual early ossification of the patella.

**Figure 5.**
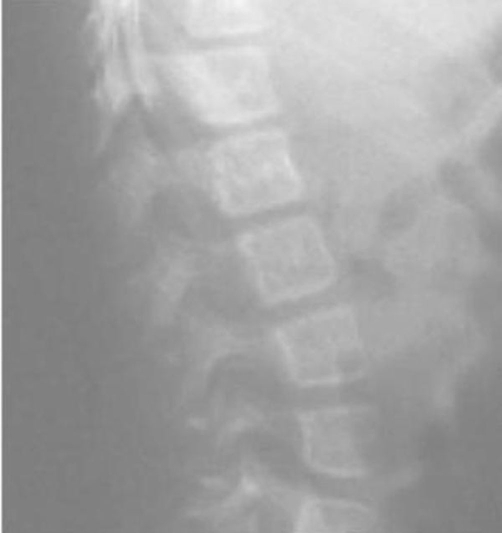
A-P spine lateral radiograph showed square shaped vertebrae.

Clinical examination at the age of 3 years showed, growth deficiency, height was 73 cm (-3SD), weight was 8 kg (-3SD) and ofc was around the 25^th^ Percentile. Craniofacially, the child showed unusual round facies, deep-seated eyes, synophyrs, curly eyelashes, flat nasal bridge, small nose, and relative prognathism (Figure 1). Short neck with apparent ptyrigium colli was notable. Musculo-skeletal examination revealed marked ligamentous hyperlaxity, normal thorax, and discrete thoraco-lumbar scoliosis. The hands were small with ulnar deviation and showed broad and proximally based thumbs. Anteroposterior radiograph of the hand showed multiple carpal ossification centres equivalent to bone age of 7 years 4 months. In addition supernumerary phalange characteristically situated between the metacarpal and proximal phalanx of the index finger, and existence of metaphyseal enlargement and pseudoepiphysis (arrow) were noted as well (Figure 2). Anteroposterior radiograph of the pelvis and proximal femur showed horizontal acetabulae, coxa vara, and short femoral necks with prominence of the lesser trochanters producing the “monkey wrench” appearance of the proximal femur. Note the remarkable prominence of the lesser trochanters (Figure 3). Lateral radiograph of the knee showed unusual early ossification of the patella (Figure 4). Spine lateral radiograph showed square shaped vertebrae (Figure 5). Vision and hearing were normal. Laboratory studies showed normal haematological components, normal calcium, phosphorus, and alkaline phosphatase levels. Urine aminoacids and mucopolysacchoroidosis were normal, and she had a normal karyotype. Abdominal ultrasound showed mild hepatomegaly. Echo-Cardio-Doppler revealed mild pulmonary stenosis with ongoing follow-ups by the cardiologist was undertaken.

## Discussion

In 1966, Desbuquois et al., [1] reported two French sisters with severe disproportionate dwarfism, joint laxity and dislocations, and disorganised ossification of the hands and feet. Particularly of significance are the unique radiological features, which included supernumerary ossification centre at the base of the proximal phalanx of the index finger and the monkey wrench (Swedish key) configuration of the femoral necks. The monkey wrench appearance of the femoral neck first emerged as a prominence of the lesser trochanter before the typical spur developed [1-9]. Mesomelic shortness, prenatal growth deficiency, marked joint laxity as well as kyphoscoliosis and multiple joint dislocations. The limb shortening affecting all segments, flaring of metaphyses of long bones, marked defective ossification of the proximal epiphyses of long bones in contrast to advanced ossification of carpal/tarsal bones. Faivre et al., [4] reported on the follow-up of 4 patients. All were obese and the facial characteristics, although still present, were less obvious. Other complications included, scoliosis, marked lordosis, coxa vara or valga and walking difficulties.

Faivre et al., [5] divided their 35 patients into 2 groups - those with the classical hand malformation (an extra ossification centre distal to the second metacarpal or a delta phalanx of the thumb) and those with normal hands.

Le Merrer et al., [8] suggested the homogeneity of the Desbuquois syndrome despite the variable expression. Hall [2] points out that death in early infancy can be common in the classical form of the condition.

Shohat et al., [9] described seven cases and provided a good review. They point out the similarity of the hand changes to those seen in Catel-Manzke syndrome. The key features in recognising children with Catel-Manzke syndrome are micrognathia, cleft palate, glossoptosis and an accessory (usually triangular) bone at the base of the index finger. Clinically the children have the appearance of severe Pierre Robin Syndrome with Hyperphalangy and Clinodactyly.

Nishimura et al., [10] reported a 14-year-old boy with radiological features of the condition (monkey wrench proximal femur, osteopenia, advanced carpal ossification) but with normal birth length, no facial dysmorphism, no radial deviation of the second digits and mild joint laxity. Height at 4 years was -5SD. Ogle et al., [11] reported two cases with obstructive sleep apnoea. One case had a marked cervical kyphosis.

Al Kaissi et al., [12] described three siblings with a rare assortment of clinical and radiological features of normal facies, normal hands, severe kyphoscoliosis, and multiple large joint dislocations. The overall clinical and radiographic phenotypes closely resembled Desbuquois dysplasia. Desbuquois dysplasia locus has been mapped to chromosome 17q25.3 [3].

Synophyrs, curly/curved and or prominent eyelashes, small short nose, and congenital cardiac defects are a constellation of features might be seen in Cornelia de Lange syndrome [13]. The overall clinico-radiographic features do not fit our patient. The phenotype of our patient could be akin to Catel-Manzke syndrome or Larsen syndrome [9,14]. In either case, however, there is absence of the “monkey wrench” configuration of the proximal femurs. Diastrophic dysplasia [6] is another clinical entity that might mimic Desbuquois dysplasia. Bieganski et al., [16] reported two sisters with severe diastrophic dysplasia, primary kyphosis and “monkey wrench” appearance and absent patellae. Our patient illustrates different phenotypic and radiographic features. Interestingly precocious ossification of the patellae is not a feature in connection with diastrophic dysplasia. Other syndromes with multiple joint dislocations have been considered in the differential diagnosis such as spondylo-epi-metaphyseal dysplasia with joint laxity [17], and spondylo-epi-metaphyseal with multiple joint dislocations [18]. Neither of these conditions fit the patient reported here.

## Conclusion

Desbuquois dysplasia is a syndromic entity reminiscent to Larsen syndrome, in that there is joint laxity with multiple dislocations. The eyes are prominent, the nasal bridge tends to be flat, and there can be marked micrognathia. Radiological changes are distinctive. Finally we wish to stress that our current report might represent the first patient from Austria with Desbuquois syndrome. Consanguinity in our family supports autosomal recessive inheritance.

## Abbreviations

MRI: magnetic resonance imaging
SD: standard deviation
